# Glass Fiber Reinforced Polymer (GFRP) Bars for Enhancing the Flexural Performance of RC Beams Using Side-NSM Technique

**DOI:** 10.3390/polym9050180

**Published:** 2017-05-19

**Authors:** Md. Akter Hosen, U. Johnson Alengaram, Mohd Zamin Jumaat, N. H. Ramli Sulong, Kh. Mahfuz ud Darain

**Affiliations:** 1 Centre for Innovative Construction Technology (CICT), Department of Civil Engineering, Faculty of Engineering, University of Malaya, Kuala Lumpur 50603, Malaysia; zamin@um.edu.my (M.Z.J.); hafizah_ramli@um.edu.my (N.H.R.S.); 2 Department of Civil Engineering, Dhaka University of Engineering and Technology (DUET), Gazipur 1700, Bangladesh; 3 Architecture Discipline, Science, Engineering and Technology School, Khulna University, Khulna 9208, Bangladesh; khmahfuz@gmail.com

**Keywords:** flexural capacity, SNSM technique, GFRP, energy absorption, ductility, stiffness

## Abstract

Reinforced concrete (RC) structures require strengthening for numerous factors, such as increased load, modification of the structural systems, structural upgrade or errors in the design and construction stages. The side near-surface mounted (SNSM) strengthening technique with glass fiber-reinforced polymer (GFRP) bars is a relatively new emerging technique for enhancing the flexural capacities of existing RC elements. Nine RC rectangular beams were flexurally strengthened with this technique and tested under four-point bending loads until failure. The main goal of this study is to optimize the structural capacity of the RC beams by varying the amount of strengthening reinforcement and bond length. The experimental test results showed that strengthening with SNSM GFRP bars significantly enhanced the flexural responses of the specimens compared with the control specimen. The first cracking and ultimate loads, energy absorption capacities, ductility and stiffness were remarkably enhanced by the SNSM technique. It was also confirmed that the bond length of the strengthened reinforcement greatly influences the energy absorption capacities, ductility and stiffness. The effect of the bond length on these properties is more significant compared to the amount of strengthening reinforcement.

## 1. Introduction

Rehabilitation or strengthening of civil engineering infrastructure has gained significant attention due to deterioration problems of structures and meeting up-to-date design requirements [1]. Numerous materials and methods have been used for strengthening structural elements. The most frequently-utilized materials for structural strengthening are steel plate and fiber-reinforced polymer (FRP). There are three types of FRP (carbon, glass and aramid) used for strengthening of structures [2]. The external bonding reinforcement (EBR) and near surface mounted (NSM) techniques are very popular for strengthening or upgrading of structural members [3,4,5,6]. The strengthening plates or laminates are glued on the tension face of the reinforced concrete (RC) beams in the EBR technique [7]. The debonding failure of the strengthening beams due to the high shear stress at the plate end [8] and corrosion of the plates due to the environmental effects [9] are some of the main concerns of the EBR technique. The intermediate crack debonding is a drawback of the EBR technique due to the frequent debonding failure mode compared to the end failure of the laminated end for beams that are well anchored [10].

In the NSM technique, the strengthening reinforcements are inserted into the concrete cover by making grooves and then filling up the grooves with adhesive or cement grout [11]. However, in this technique, the main drawback is the premature debonding of the strengthening reinforcements and concrete cover separation. Moreover, the NSM technique delays the debonding failure in comparison to the EBR technique [3]. Tang et al. [12] studied the performance of RC normal and lightweight polystyrene aggregate concrete beams that were flexurally strengthened using NSM GFRP bars. The dominant failure mode of these strengthened beams was debonding. Reda et al. [13] studied the flexural performance of NSM-GFRP bar-strengthened RC beams using different end-anchorage conditions. The NSM technique using GFRP bars and end-anchorage revealed higher stiffness and improved failure modes compared to strengthened beam without end anchorage. Escórcio et al. [14] investigated the experimental behavior of RC beams’ main steel rebars replaced with GFRP bars as a rehabilitation of corroded reinforced concrete beams. GFRP bar-rehabilitated beams exhibited higher flexural capacity and less deflection compared with the original RC beam. Almusallam et al. [15] conducted the experimental and numerical investigation on the RC beams that were flexurally strengthened by NSM steel/GFRP bars. Most of the strengthened beams failed due to the yielding of internal steel bars and the crushing of compression concrete. Jung et al. [16] studied the flexural behavior of strengthened RC beams with the NSM and EBR technique using CFRP reinforcement. The NSM CFRP bars and EBR-strengthened specimens failed by debonding, while mechanical interlocking grooves were used to eliminate debonding. Al-Mahmoud et al. [17] and Kalayci et al. [18] carried out an experimental program to evaluate the flexural strength of RC beams strengthened with NSM FRP bars, and they reported premature debonding failure of the strengthened beams. Soliman et al. [19] investigated the behavior of RC beams flexurally strengthened with NSM using different bond lengths, diameters and types of FRP bars. The test results indicated that the application of NSM FRP bars was useful for improving the flexural strength, and all strengthened beams failed by concrete cover splitting. 

Sharaky et al. [20,21] investigated the flexural behavior of NSM-strengthened RC beams by applying FRP (carbon and glass) bars or strips. The test variables were the diameter and the number of NSM bars and various types of NSM reinforcement and epoxy. The strengthened specimens failed by premature debonding, i.e., concrete and epoxy interface failure, epoxy splitting and concrete splitting or cover separation. Hosen et al. [22] proposed the innovative side near-surface mounted (SNSM) technique to mitigate the problem (overlapping stress and concrete cover separation) in the NSM technique for flexurally-strengthened RC beams. In this study, different diameters of steel and CFRP-strengthened bars were used for experimental and analytical investigation. The test results revealed that the SNSM technique significantly improved the flexural responses and serviceability of the RC beams. Shukri et al. [23] investigated the flexural behavior of pre-cracked RC beams strengthened by the SNSM technique using CFRP bars, and the experimental results were verified by the moment-rotation approach. The pre-cracked specimens show the same failure modes, but had higher stiffness compared to the non-pre-cracked specimens. 

From the literature, it is revealed that the SNSM technique with GFRP bars has rarely been incorporated. The aim of this paper is to further investigate the flexural behavior of RC beams strengthened by the SNSM technique using GFRP bars. The effect of SNSM-GFRP bars on the load carrying capacity, deflection, failure mode, energy absorption capacity and ductility and stiffness was examined and compared with the control specimens. Moreover, the influence of the amount of strengthening reinforcement and bond length on the structural performance was also assessed. 

## 2. Experimental Procedure

The investigation on the behavior of RC beams strengthened with the side near surface mounted (SNSM) technique using glass fiber-reinforced polymer (GFRP) bars was carried out. The variables investigated include strengthened bar diameter and bonded length, as shown in Table 1. 

### 2.1. Concrete and Steel

The ready mixed concrete was used to cast the beam specimens, prisms, cylinders and cubes. Crushed granite aggregates of 20 mm maximum size were used as the coarse aggregate. The compressive strength was determined in accordance with BS EN 12390-3 [24] using three 100 mm cube specimens, resulting in an average compressive strength of ~26 MPa. The modulus of rupture and splitting tensile strength tests were carried out based on BS EN 12390-5 [25] and BS EN 12390-6 [26], respectively; average values of ~2.71 MPa and 3.86 were obtained for the splitting tensile strength and modulus of rupture, respectively. The modulus of elasticity (MOE) value of 28.50 GPa was obtained for the concrete tested as per ASTM C469 [27].

The reinforcement cage was prepared using tensile reinforcement of 12 mm Ø deformed steel reinforcing bars with a yield strength of 550 MPa. For the compression reinforcement, 10 mm Ø deformed steel with a yield strength of 520 MPa was used. Plain mild steel bars of 6 mm Ø of Grade 300 were used as stirrups. The modulus of elasticity of the reinforcement was 200 GPa.

### 2.2. GFRP Reinforcements

**Figure 1:**
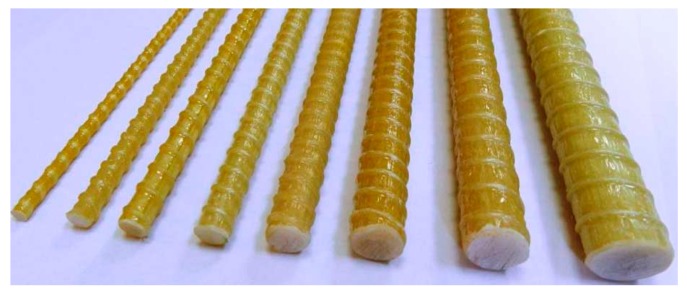
GFRP bars.

The ribbed GFRP bars (Figure 1) were used for the RC beam specimens flexurally strengthened by the SNSM techniques. Based on the manufacturer’s information (Haining Anjie Composite), the density, tensile and shear strength and modulus of elasticity of the GFRP bars are given in Table 2. 

### 2.3. Epoxy Adhesive

Sikadur^®^ 30 epoxy paste adhesive was used as a bonding agent between the strengthening reinforcements and concrete substrate of the specimens [28]. The epoxy adhesive has Components A and B; A is white in color and consists of the epoxy resin, while B is black in color and consists of a hardener. The two components were mixed at a ratio of 3:1 until a consistent gray color was achieved. 

### 2.4. Specimen Preparation and Strengthening Procedure

**Figure 2:**
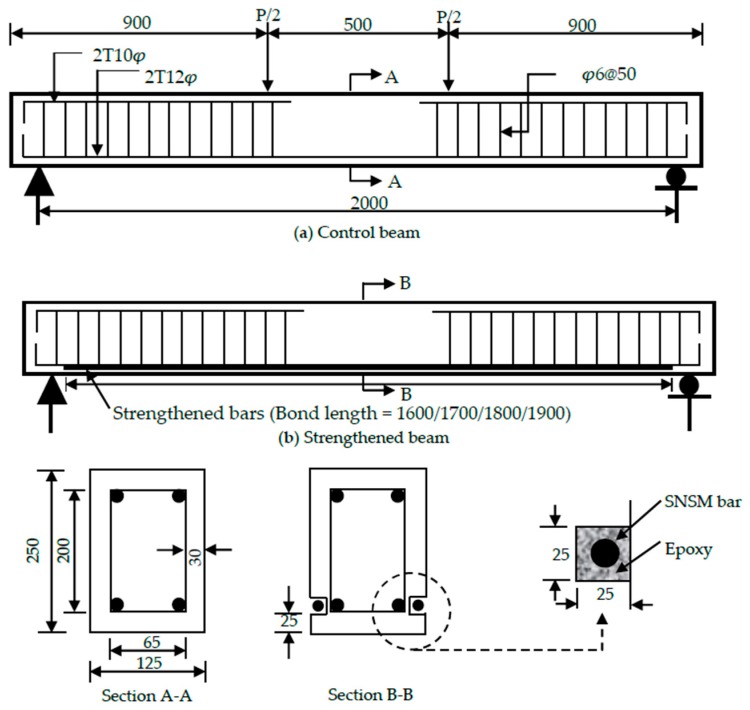
Details of the beam specimens (dimensions in mm).

A total of nine RC rectangular beam specimens was prepared and tested. The cross-section of the specimens was 125 mm × 250 mm, total span length 2300 mm, clear span length 2000 mm and a shear span length of 750 mm, as shown in Figure 2. The 12-mm bars were used as tension reinforcement with both ends bent (90°) to fulfill the anchorage criteria. The 10-mm bars were used as hanger bars up to the shear span zone, and 6-mm bars were used for stirrups.

The SNSM strengthening technique was used in this study. In this technique, strengthening bars were installed into grooves in a longitudinal direction on both sides of the specimens. The grooves were cut in the concrete cover using a special concrete saw with a diamond blade. The dimensions of the grooves are shown in Figure 2. The residual concrete lugs from the lower surface of the grooves were removed by a hammer and hand chisel. Finally, the grooves were cleaned with acetone and a high pressure air jet.

**Figure 3:**
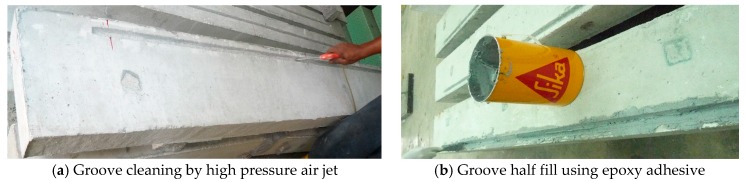

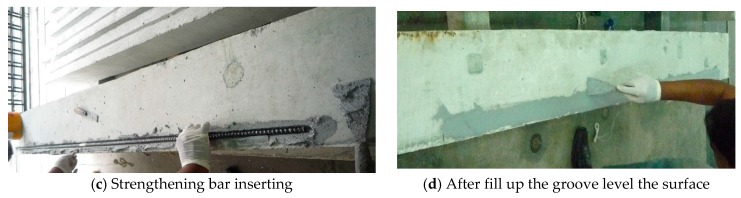
SNSM technique strengthening procedure.

The grooves were half-filled using epoxy adhesive and strengthening GFRP bars placed into the grooves with slight force as shown in Figure 3. This force caused the adhesive to flow around the strengthening GFRP bar. More adhesive was used to fill up the groove and level the surface. The strengthened specimens were stored to harden for a week and cure the epoxy adhesive to attain full strength. 

### 2.5. Experimental Set-Up

**Figure 4:**
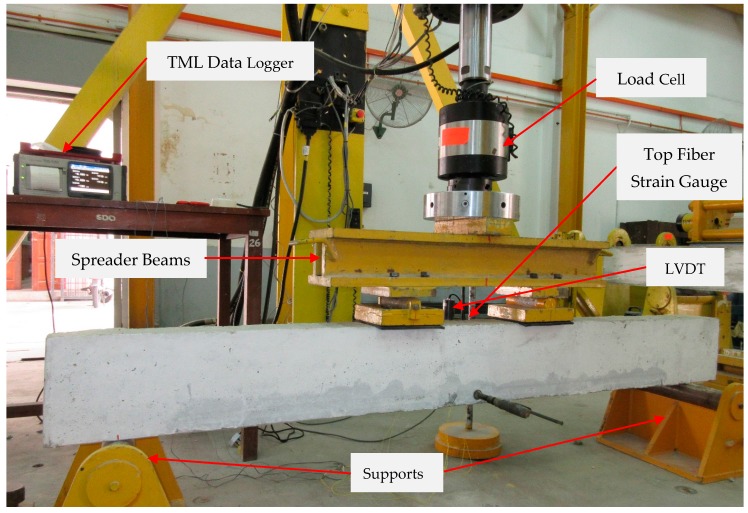
Laboratory loading setup. LVDT, linear variable differential transducer.

All of the RC beam specimens (control and strengthened) were tested under four-point bending until failure using an Instron Universal Testing Machine, as shown in Figure 4. One vertical linear variable differential transducer (LVDT) was placed at the mid-span of the beam to measure the deflection and ensure that the transducer touched the bottom face of the specimen. The compressive strains of the top surface of the concrete specimens were measured using a 30-mm strain gauge that was affixed at mid-span of the beam specimen. A 30-mm strain gauge was fixed at the center of the strengthening bar using Araldite epoxy adhesive to measure the tensile strain of the strengthening reinforcement. All of the tests were carried out under displacement control with the rate of the actuator set at 1.5 mm/min. All of the data were recorded at 10-s intervals using a TDS-530 data logger. The crack width of beam specimens was measured by a Dino-Lite digital microscope.

## 3. Test Results and Discussion

### 3.1. First Cracking and Ultimate Load Capacities

**Figure 5:**
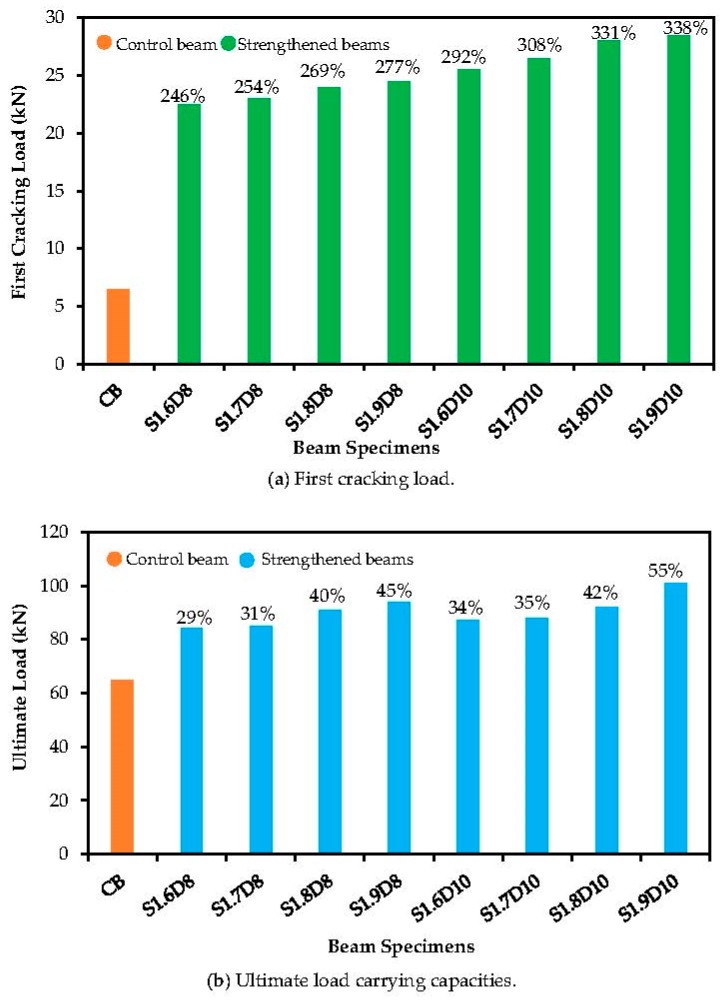
Flexural capacities improved by SNSM-GFRP bars.

The first cracking and ultimate load carrying capacities of the beam specimens are shown in Figure 4. The RC specimens strengthened with the SNSM technique using GFRP bars reported a significant increase of their stiffness at the pre-cracking phase (Figure 5a). The first crack load is very important, as the stiffness of the beam decreases after the formation of the first crack [29]. The beams with GFRP bars improved the first cracking load to up to 4.38-times compared with the control beam. As the bond length of the strengthened reinforcements was increased from 1600 to 1900 mm, it improved the first cracking loads by 46% and 31%, respectively, for the 10 mm Ø and 8 mm Ø bars. 

The beam specimens strengthened with steel and CFRP bars enhanced the first cracking load up to 3.17- and 2.02-times, respectively, over the control specimen by the SNSM technique [22]. The RC beams strengthened with the near surface mounted technique (NSM) using CFRP bars increased the first cracking load up to 76% [17]. However, RC beams that were strengthened using GFRP bars by the NSM technique reported an increase of their first cracking load of up to 56% [13]. 

Moreover, the first crack load of the RC beams flexurally strengthened with NSM steel or GFRP bars [15] compared with lightweight concrete beams strengthened with NSM-GFRP bars [12] did not show much difference in the context of the first crack load. 

The beams strengthened with SNSM-GFRP bars resulted in an efficient increase of the ultimate load carrying capacity, as shown in Figure 5b. The SNSM technique with a 1900-mm bond length of 8 mm Ø and 10 mm Ø GFRP bars increased the ultimate load up to 45% and 55%, respectively, compared with the control specimen. Increasing the bond length of 8 mm Ø and 10 mm Ø GFRP bars from 1600 mm to 1900 mm resulted in an increase in the ultimate loads by 16% and 21% respectively, over the control specimen. 

However, the NSM-GFRP bars with end anchorage resulted in an enhanced ultimate load carrying capacity of up to 101% compared with the control beam [13]. The NSM-steel and NSM-GFRP increased the ultimate load till 30% and 24%, respectively [15]. By contrast, the lightweight concrete beams with NSM-GFRP bars increased till 53% [12] and RC beams with prestressed NSM-CFRP bars increased three to four times [30].

### 3.2. Load-Deflection Behavior

**Figure 6:**
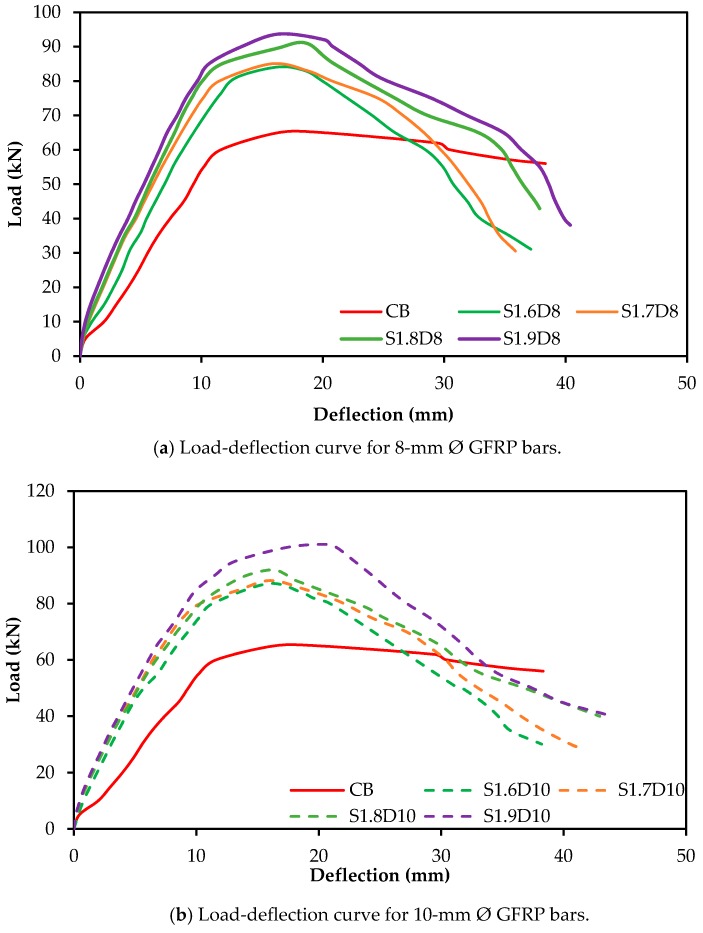
Load-deflection curves for tested beam specimens.

**Figure 7:**
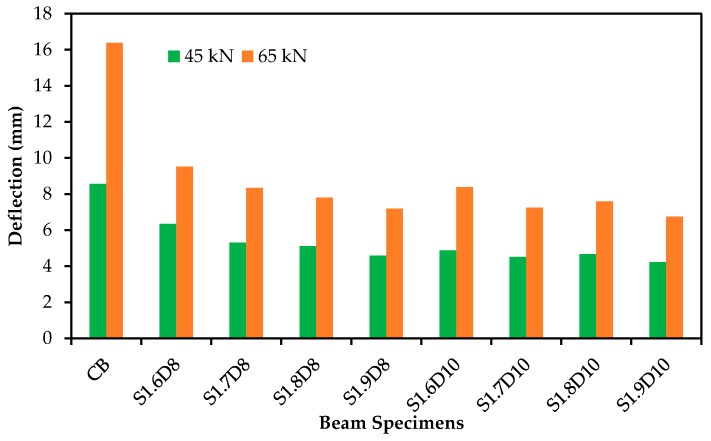
Deflection at different loads (45 and 65 kN).

The load versus deflection curves for RC beam specimens strengthened with SNSM-GFRP bars are shown in Figure 6. As seen in the figure, the load-deflection curves exhibit tri-linear stages, as per the usual failure mechanism, which is followed by cracking of concrete to reinforcement yielding, yielding to ultimate and ultimate to failure. The behavior of all strengthened specimens followed linear and elastic patterns in the first stage, due to the full flexural rigidity of the beam. The SNSM-GFRP bars greatly influence the load-deflection curves over the control beam in the first stage. The second stage starts from the yielding of the tension reinforcement, at this stage, SNSM bars control the number and width of cracks, as the maximum tensile stress of concrete exceeded the flexural strength of concrete. The rate of increasing deflection was found to be higher than the previous stage, which resulted in decreased stiffness of the beam. The final stage is the ultimate load to failure of the specimen. At this stage, the load gradually reduced and rapidly increased the deflection due to the linear stress-strain characteristics of the GFRP bars [31], and the SNSM grooves’ epoxy adhesive cracking occurred after the tension steel reinforcement yielding. Figure 7 shows the reduction of deflection due to strengthening by the SNSM technique using GFRP bars (at 45-kN and 65-kN applied loads). The deflection decreased by about 46% and 56% for 8 mm, and 51% and 59% for 10-mm SNSM-GFRP bars at 45 and 65 kN, respectively. 

However, the RC beams flexural strengthened with externally-bonded carbon fiber-reinforced polymer (CFRP) sheets [32] or strengthened with near surface mounted CFRP bars [33] show different patterns of load-deflection curves. 

### 3.3. Cracking Behaviors

As is known, concrete cracks when its tensile stress exceeds the limiting tensile strength of the designated concrete [34]. The crack formation and propagation in concrete depends on the tensile strength. When the principal tensile stress in the beams exceeds the concrete tensile strength, flexural cracks occur in the vertical direction [35]. 

**Figure 8:**
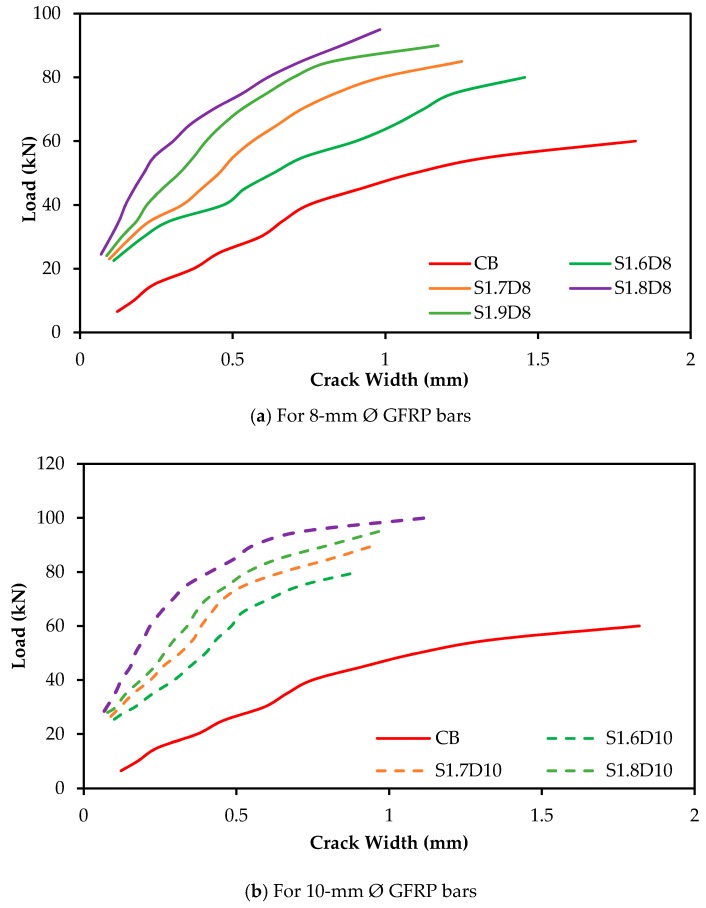
Load-crack width of the specimens.

**Figure 9:**
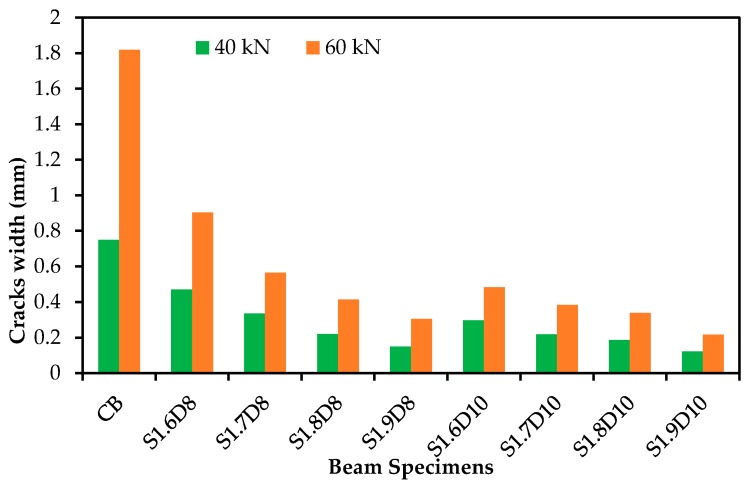
Cracks width of the specimens at 40 and 60 KN.

The load versus crack widths of the beam specimens are shown in Figure 8. The crack width of the specimens was measured beyond their yield load, which might be close to the ultimate load carrying capacity of the specimen using a Dino-Lite digital microscope within the location of the main reinforcement at constant moment zones at various load levels. The trend of the curves of the 8-mm Ø GFRP-strengthened specimens was almost linear, while the 10-mm Ø curves are steep compared with the control specimen. The crack widths were significantly reduced by ~80% and 83% for 8-mm Ø and 84% and 88% for 10-mm Ø SNSM-GFRP bars at 40 kN and 60 kN, respectively, for a fixed bond length of 1900 mm (Figure 9). Thus, all of the GFRP-strengthened specimens reported reduced crack widths at all load levels compared with the control specimen, which is attributed to the increased stiffness of the beams due to the GFRP bars. The total number of cracks and the average crack spacing for beams CB, S1.6D8, S1.7D8, S1.8D8, S1.9D8, S1.6D10, S1.7D10, S1.8D10 and S1.9D10 were 18, 24, 26, 25, 28, 30, 27, 29 and 32; and 92, 72, 67, 65, 61, 60, 63, 56 and 52 mm, respectively. Thus, as expected, the strengthening by SNSM-GFRP has a significant effect, as it increased the number of cracks and subsequently reduced the average crack spacing of the specimens. 

### 3.4. Failure Modes

**Figure 10:**
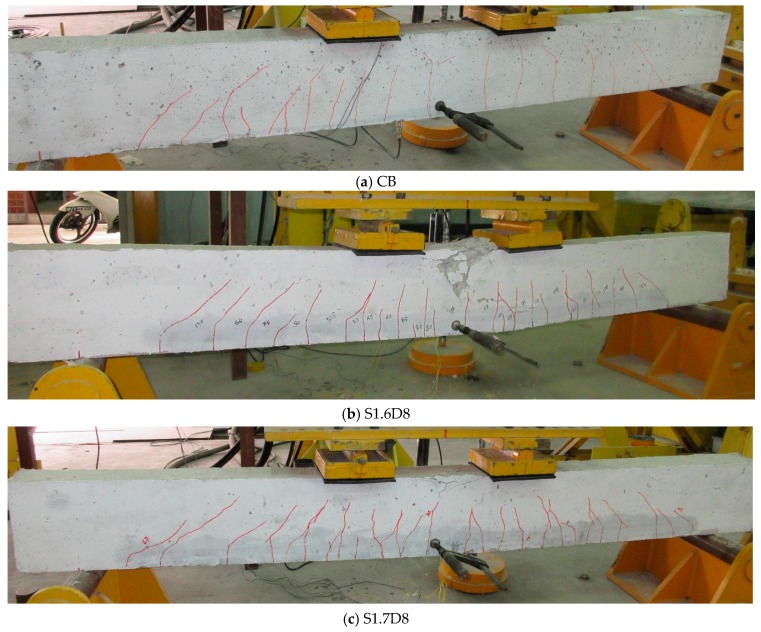

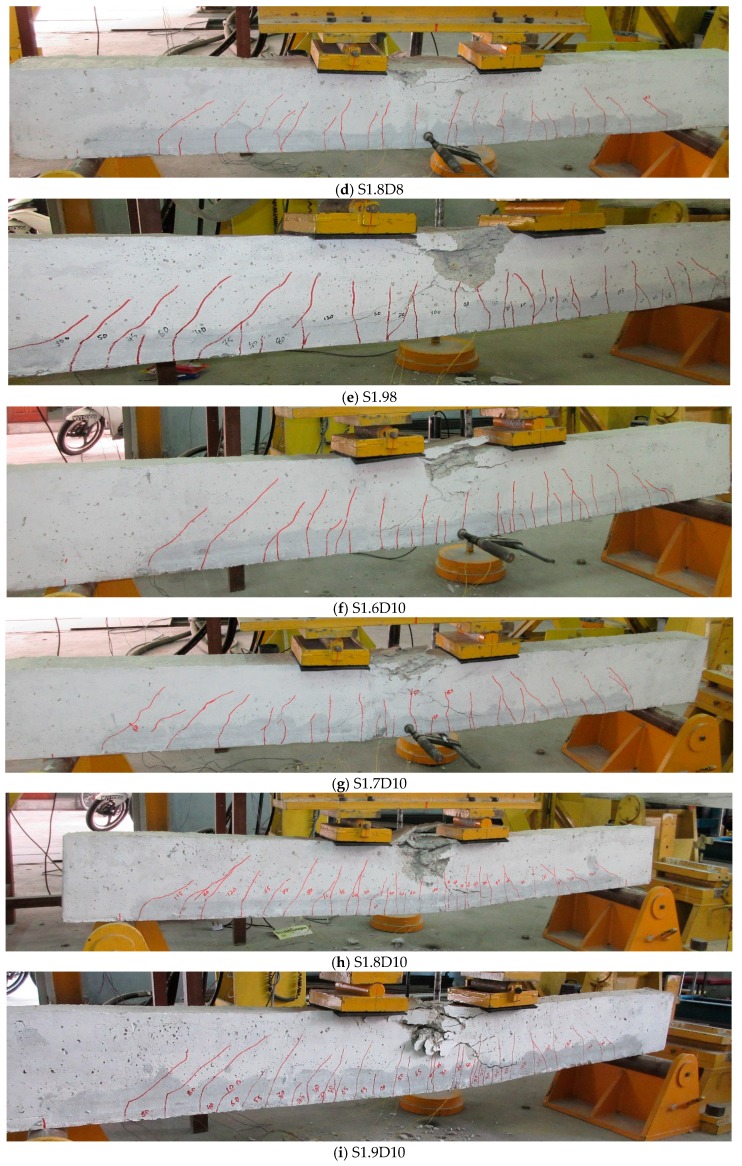
Failure mode of the specimens.

The failure modes of the control and all SNSM-GFRP-strengthened beam specimens are shown in Figure 10. The specimens reported a very similar failure mode, i.e., flexural failure, yielding of tensile steel, followed by crushing of the concrete in the compression zone. First, a fine flexural crack was developed at mid-span and gradually propagated towards the neutral axis of the specimen. Further flexural cracks were developed and continued to widen as the load increased; however, the presence of SNSM-GFRP bars controlled the crack width up till failure of the strengthened specimens compared with the control specimen. The leading flexural cracks began and propagated along the depth of the specimen section at the maximum bending moment region. Once those cracks were extended to nearly the full depth of the section, then the specimen fails. Few shear cracks were initiated between the spreader loading point and support of the specimen; however, final failure of the specimen was not affected by those shear cracks. 

The final failure of the strengthened specimens was flexure and crushing of concrete at top-most compression zone of the section. It is the most momentous mode of failure in SNSM-GFRP-strengthened specimens in contrast to the specimens strengthened using NSM-FRP with different bond lengths, which had concrete covering the separation failure modes [19]. The NSM-FRP-strengthened RC beams failed by debonding of the FRP reinforcement and epoxy adhesive [16]. Most of the NSM strengthened beams with small embedment length of CFRP strips failed via debonding [36]. Hence, the bond performance of SNSM-GFRP bars exceeds that of NSM-FRP bars to concrete. 

### 3.5. Energy Absorption Capacity

**Figure 11:**
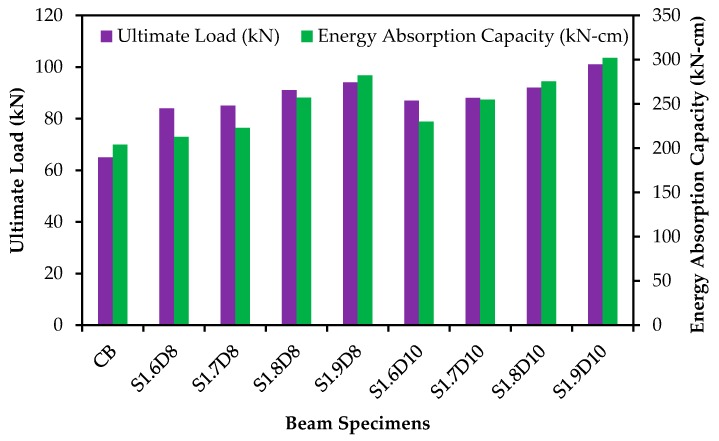
Ultimate load and energy absorption capacities of the specimens.

The energy absorption capacity is an essential structural property of RC elements, while existing structures are repaired, strengthened or upgraded with strengthening materials or techniques [37]. The energy absorption capacity is defined as the energy absorbed by the unit cross-sectional area of the specimens computed at any displacement terminal point [38,39]. The energy absorption capacity was determined using the area of the load versus deflection curve (at mid-span) up to the failure of the specimens. The ultimate load and energy absorption capacities of the specimens are demonstrated in Figure 11. The use of SNSM-GFRP 8-mm Ø and 10-mm Ø bars shows an improvement in the energy absorption capacity of up to 38% and 48%, respectively, compared with the control specimen. Increasing the amount and bond length of the strengthening reinforcement of GFRP progressively enhanced the ultimate load and energy absorption capacities. The SNSM-GFRP bars carry the loads up to the failure of the beams. 

The energy absorption capacity was reduced by ~49%, while the NSM technique was used to strengthen RC beams using FRP bars [21]. 

### 3.6. Ductility

The ductility of an RC beam can be defined as its capability to endure inelastic deformation without reduction of load carrying capacity before failure [40]. The significant aspect of ductility of any structures is a precaution in advance of failure. Ductile RC structures provides ample warning before failure, whereas for brittle structures, it provides little or no warning prior to failure [41]. The deflection ductility index is attained [42] from load-deflection diagram of the specimens using the following equations.

(1) $$\mu_{\Delta u}=\frac{\Delta_{u}}{\Delta_{y}}$$

(2) $$\mu_{\Delta f}=\frac{\Delta_{f}}{\Delta_{y}}$$

where $\mu_{\Delta u}$ and $\mu_{\Delta f}$ are the deflection ductility index at maximum load and at failure load, respectively, and $\Delta_{u}$, $\Delta_{f}$ and $\Delta_{y}$ are the deflection at the maximum load, failure load and yield load, respectively. The ductility index at ultimate and failure loads and the comparison with the control beams are presented in Table 3. The ductility index at ultimate and failure loads shows an improvement with increasing bond length of the strengthened GFRP bars. Furthermore, the increasing amount of strengthening reinforcement significantly enhanced the ductility due to flexure failure mode and the low Young’s modulus of the elasticity of GFRP bars [43]. Hence, the beam strengthened with SNSM-GFRP bars is very advantageous in terms of ductility. Therefore, all specimens strengthened with SNSM-GFRP bars reported adequate ductility, as confirmed by the ductile mode of failure.

However, the ductility decreased with increasing amounts of strengthening reinforcements when the RC beam was strengthened with EBR [44] or NSM [45] techniques using FRP.

### 3.7. Stiffness of the Beams

Stiffness is defined as the capability to prevent bending or deflection of the specimens under loading. It is one of the most important characteristics of the RC structures under serviceability behavior [46]. Parameters, such as cracks patterns, displacement and ductility, are influenced by stiffness. The stiffness of the specimens assessed from the gradient of the load-deflection curve at service load level is shown in Figure 6. A deflection of about 4 mm of the control specimen was determined to be equal to the effective span/480 at the service load [47]. Therefore, the service loads for all strengthened specimens were measured at a reference deflection of 4 mm. The stiffness of the specimens was evaluated as the ratio of the service load to the corresponding deflection.

**Figure 12:**
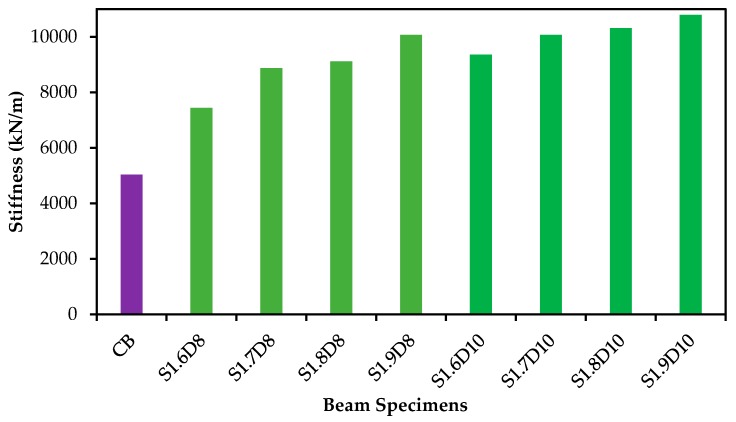
Stiffness of the beam specimens.

The stiffness at service load of the beam specimen is graphically presented in Figure 12. The SNSM-GFRP-strengthened specimens resulted in higher stiffness compared with the control specimen. Increasing the size of GFRP bars from 8 to 10 mm Ø increased the stiffness from 100% to 114% compared with the control specimen. Moreover, the increase in the bond length from 1600 mm to 1900 mm improved the stiffness by ~35% and 15%, respectively, for 8-mm Ø to 10-mm Ø GFRP bars. In this study, the stiffness of the strengthened specimens mostly depended on the size of the SNSM bars. 

### 3.8. Effect of Bond Length

**Figure 13:**
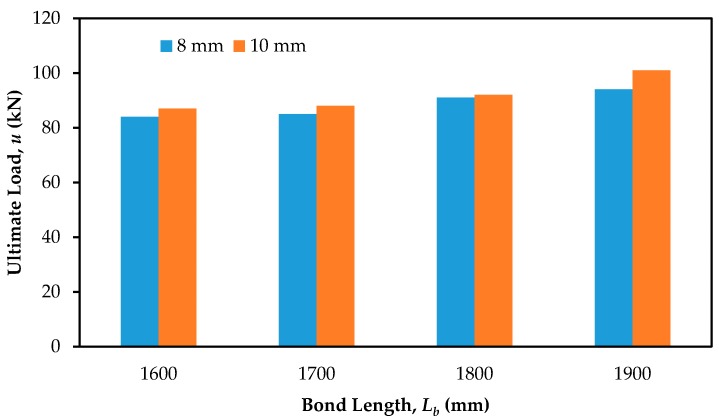
The effect of bond length on the ultimate load.

**Figure 14:**
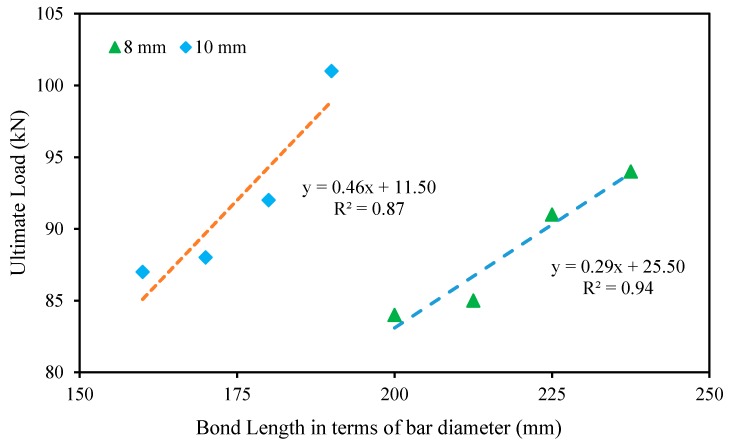
Relationship between the bond length and ultimate load carrying capacity.

The effect of bond length of the strengthened bars on the ultimate load is presented in Figure 13. Based on the experimental results, by increasing the bond length from 1600 to 1900 mm, the ultimate load carrying capacity increased from 29% to 45% and 34% to 55% compared with the control specimen with 8-mm Ø and 10-mm Ø GFRP bars, respectively. Thus, the bond length of the strengthened bars enhanced the flexural performance of the specimen. Increasing the bond length reduced the distance between the bar and the support, which is an influential parameter in concrete cover separation of NSM-steel/FRP-strengthened beams [48]. The relationship between the bond length and ultimate load carrying capacities of the strengthened beam specimens was correlated and shown in Figure 14, while the equations for the relationship are as follows:

*L_b_* = 0.46*u* + 11.5 (for 10 mm strengthened bars) (3)

*L_b_* = 0.29*u* + 25.5 (for 8 mm strengthened bars) (4)

where *L_b_* = bond length in terms of bar diameter (mm) and *u* = ultimate load (kN).

### 3.9. Effect of Amount of GFRP Reinforcement

**Figure 15:**
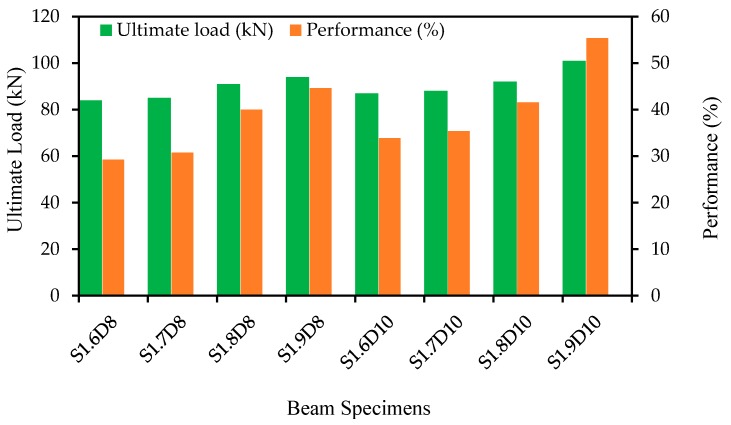
The effect of strengthening reinforcements on the flexural performance.

Figure 15 shows the effect of strengthening reinforcements on the flexural performance [Performance = {(ultimate load of strengthened beam − ultimate load of control beam)/ultimate load of control beam} × 100] of the beams strengthened with the SNSM technique using GFRP bars. The amount of reinforcement has significant influence on the flexural capacity of the normal RC beams [49]. In the SNSM technique, an increase in the amount of strengthening reinforcement reduces the amount of epoxy adhesive, thereby affecting the performance of the bond between the GFRP bars and concrete [50]. Therefore, it is important to investigate the effect of the amount of reinforcements on the performance of strengthened beams. The flexural performance increased from 45% to 55% with the increase in the amount of strengthening reinforcements from 50 to 79 mm^2^ for a fixed bond length of 1900 mm.

### 3.10. Main Steel Reinforcement Tensile Strain of the Specimens

**Figure 16:**
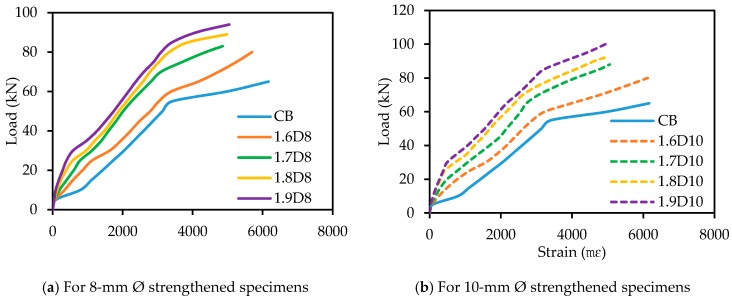
Tensile strain in the internal main steel reinforcement.

Figure 16 shows the load versus tensile strains of the main steel reinforcement. The tensile strains in the steel bars of all of the strengthened beams showed higher strains compared with the strains in the tension steel of the control specimen due to the greater stiffness of the strengthened beam specimens. While the first flexural crack occurs in the concrete section, an abrupt increase of tensile strain in the steel bars due to the tensile stresses was shifted to the internal main steel reinforcement of the specimens. The rate of increment for strengthened beam specimens was lower than the control specimen, because of the bigger flexural crack width of the control specimen. The tensile strains of the beam specimens exhibited almost a linear behavior from the first cracking to the yielding of the steel reinforcement. After yielding of the reinforcement, strains in the steel bars were rapidly increased.

### 3.11. Tensile Strain in the SNSM GFRP Reinforcement

**Figure 17:**
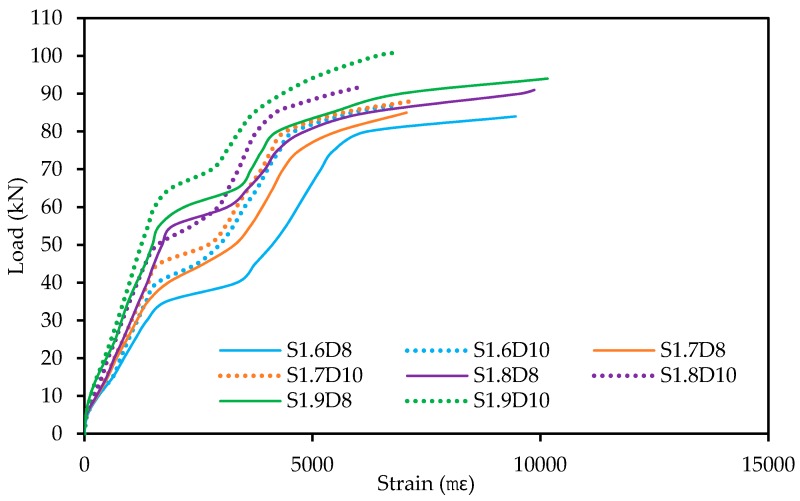
Tensile strain in the SNSM-GFRP bars.

The tensile strains of the side NSM GFRP reinforcement curves at different loads are presented in Figure 17. The tensile strains in the SNSM-GFRP strengthening bars were measured by fixing 30-mm strain gauges at the mid-span of the bars. On average, the GFRP bars followed a similar strain profile under the flexural load. The SNSM-GFRP specimens exhibited nearly linear curves up to the loads of 35, 40, 55, 55, 40, 45, 50 and 65 kN for S1.6D8, S1.7D8, S1.8D8, S1.9D8, S1.6D10, S1.7D10, S1.8D10 and S1.9D10, respectively. Upon reaching these loads, strain values in the GFRP bars abruptly increased, which may have been due to the propagation of the cracks at the positions of these strain gauges. The tensile strains in the SNSM GFRP-bars in all strengthened beam specimens increased significantly. After yielding, the SNSM bar strain of the specimens gradually increased until the failure of the specimens, and strain data were recorded until the damage of the strain gauges in the bars.

### 3.12. Moment-Curvature Behavior of the Specimens

To determine the curvature of the beam specimens due to the externally-applied experimental load, the following equation was used [51].

(5) $$\varphi =\frac{\varepsilon_{c}+\varepsilon_{s}}{d}$$

where *ϕ* is the curvature of the beam specimens, *ɛ_c_* is the extreme fiber concrete compressive strain and *ɛ*_s_ is the tensile strain of the main steel bars. 

**Figure 18:**
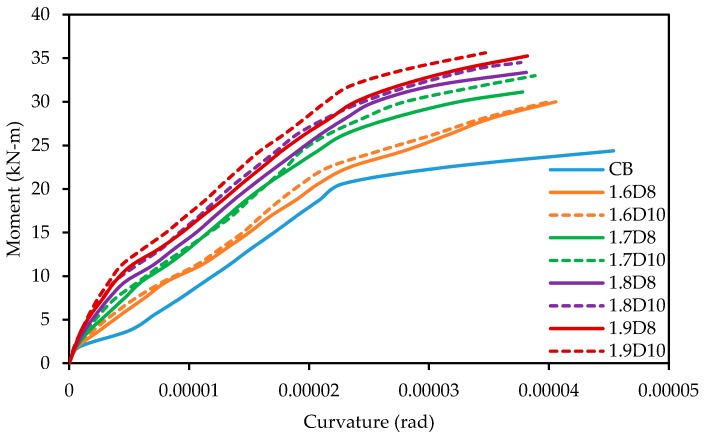
Moment-curvature relationship for all beam specimens.

Figure 18 shows the moment versus curvature relationship for control and all strengthened beam specimens. The moment-curvature curves for all beam specimens depict a trilinear behavior, which are defined by uncracked, cracked and yielded segments. The uncracked segment curves slope very steeply, representing the full flexural rigidity of the specimens. The cracked segment shows the reduction in the slope of the curvature curves due to decrease in its flexural stiffness. The final segment shown a significant increase in the curvature up to failure of the specimens. 

## 4. Analytical Study

### 4.1. Prediction of Deflection Behavior

The deflection of the strengthened beam specimens throughout the experiment depends on the stiffness, brittle-elastic characteristics of concrete and the predominant bond between the surrounding concrete and strengthened GFRP bars. The un-cracked moment of inertia is equal to its gross moment of inertia (*I*_g_ *= bh*^3^/12) of the cross-section. Once the externally-applied moment (*M*_a_) exceeds the cracking moment (*M*_cr_) of the section, then the crack occurs, and this decreases the stiffness of the section. At this phase, the moment of inertia lies between the gross moment of inertia (*I*_g_) and the cracking moment of inertia (*I*_cr_). The elastic analysis of the RC beam section was used for determining the cracking moment of inertia (*M*_cr_) as specified by the flowing equations [52].

(6) $$I_{\mathrm{cr}}=\frac{bd^{3}}{3}k^{3}+n_{\mathrm{snsm}}A_{\mathrm{snsm}}d^{2}{\left(1-k\right)}^{2}$$

(7) $$k=\sqrt{2\rho_{\mathrm{snsm}}n_{\mathrm{snsm}}+{\left(\rho_{\mathrm{snsm}}n_{\mathrm{snsm}}\right)}^{2}}-\rho_{\mathrm{snsm}}n_{\mathrm{snsm}}$$

where *b* is the width of the cross-section, *d* is the effective depth of the section, *k* is the ratio of the depth of the neutral axis (N.A.) to the reinforcement depth, *n*_snsm_ is the ratio of the modulus of elasticity of the SNSM bars to the modulus of elasticity of concrete and *A*_snsm_ is the area of SNSM bars. In this case, the moment of inertia of the beam section no longer has a constant value. Hence, the effective moment of inertia (*I*_e_) can be used for the calculation of the deflection by the Branson equation [53]. Branson’s equation was recommended by American Concrete Institute (ACI) [54]. Therefore, the effective moment of inertia and deflection (Δ) are expressed as given hereunder:

(8) $$I_{e}={\left(\frac{M_{\mathrm{cr}}}{M_{a}}\right)}^{3}I_{g}+\left[1-{\left(\frac{M_{\mathrm{cr}}}{M_{a}}\right)}^{3}\right]I_{\mathrm{cr}}\leq I_{g}$$

(9) $$\Delta =\frac{PL_{a}\left(3L^{2}-4{L_{a}}^{2}\right)}{48E_{c}I_{e}}$$

where *P* is the applied load, *L* and *L*_a_ are the effective and shear span length of the beam specimens, respectively, and *E*_c_ is the modulus of elasticity of the concrete.

### 4.2. Prediction of Flexural Crack Spacing and Width

The flexural crack spacing and width of the specimens were calculated in accordance with Eurocode 2 [55], which depends on the modular ratio of the SNSM bars, steel reinforcement, concrete cover and the location of the neutral axis (N.A.) for the composite section of the specimens. The following equations were used to evaluate the flexural crack spacing and width of the SNSM-GFRP bar-strengthened beam specimens.

(10) $$S=3.4c+0.425k_{1}k_{2}\frac{\varphi_{d}}{\rho_{\mathrm{eff}}}$$

(11) $$\rho_{\mathrm{eff}}=\frac{A_{s}+n_{\mathrm{snsm}}A_{\mathrm{snsm}}}{A_{\mathrm{eff}}}$$

(12) $$A_{\mathrm{eff}}=\min\left\{\begin{array}{l} 2.5xbxc\\ bx(h-y)/3 \end{array}\right\}$$

(13) $$w_{k}=S\left(\varepsilon_{\mathrm{sm}}-\varepsilon_{\mathrm{cm}}\right)$$

(14) $$\varepsilon_{sm}-\varepsilon_{cm}=\frac{\sigma_{s}-k_{t}\frac{f_{ct}}{\rho_{eff}}\left(1+\alpha_{e}\rho_{eff}\right)}{E_{s}}\geq 0.6\frac{\sigma_{s}}{E_{s}}$$

(15) $$\alpha_{e}=\frac{E_{s}}{E_{c}}$$

where *S* is the flexural crack spacing, *k_1_* is the bond coefficient (0.8 and 1.6 for a high bond and plain steel rebar, respectively), *k_2_* is the strain distribution coefficient (0.50 and 1.0 for bending and pure tension, respectively), *ϕ*_d_ is the diameter of the steel reinforcement, $\rho_{eff}$ is the effective reinforcement ratio, *A*_eff_ is the effective area of concrete in tension, *w_k_* is the width of crack, $\varepsilon_{sm}$ is the mean strain in the reinforcement for the effects of tension stiffening of the concrete, $\varepsilon_{cm}$ is the mean strain in the concrete between cracks, $\sigma_{s}$ is the stress of the tension reinforcement, *k*_t_ is the factor of the duration of loading (0.4 and 0.6 for long- and short-term loading, respectively), $f_{ct}$ is the tensile strength of the concrete, and the remaining symbols designate their usual meanings.

### 4.3. Verification of Load-Deflection Curves

**Figure 19:**
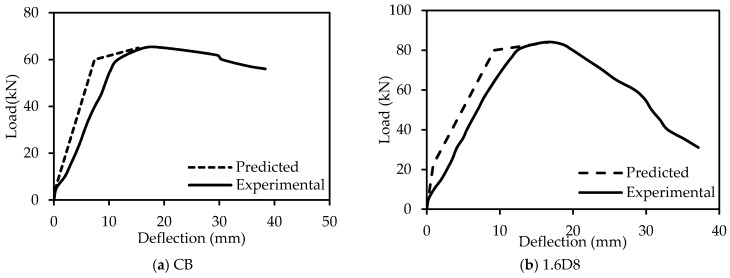

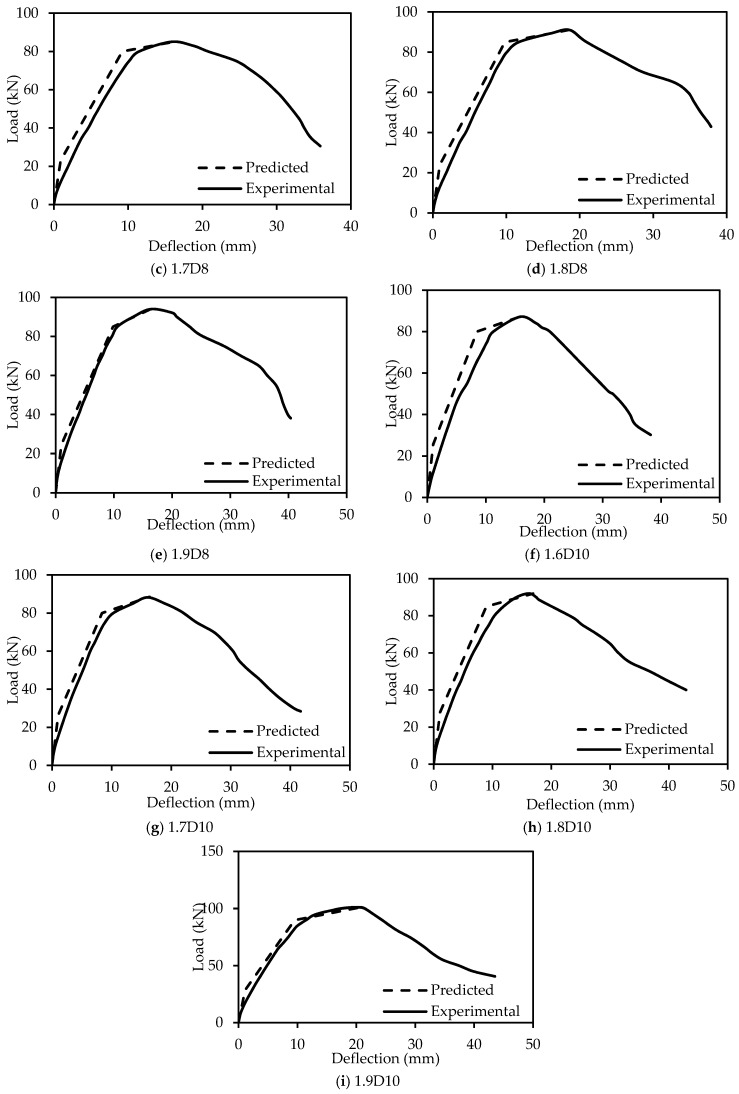
Comparison between the experimental and predicted load-deflection curves.

The comparisons between the experimental and analytically-predicted load versus deflection curves are shown in Figure 19. The experimental and predicted results show a very good agreement for control and strengthened beam specimens.

### 4.4. Verification of Flexural Crack Spacing and Width

The experimental flexural crack spacings obtained for the beam specimens were 92, 72, 67, 65, 61, 60, 63, 56 and 52 mm for S1.6D8, S1.7D8, S1.8D8, S1.9D8, S1.6D10, S1.7D10, S1.8D10 and S1.9D10, respectively. The predicted crack spacings were 152, 120 and 119 mm for the control, the specimens with 8-mm Ø and 10-mm Ø GFRP bars, respectively. The experimental and predicted crack widths were 1.82, 1.45, 1.25, 1.17, 0.98, 0.9, 0.95, 0.97 and 1.12 mm; and 0.38, 0.28, 0.29, 0.31, 0.32, 0.29, 0.3, 0.31 and 0.35 mm for the S1.6D8, S1.7D8, S1.8D8, S1.9D8, S1.6D10, S1.7D10, S1.8D10 and S1.9D10 beam specimens, respectively. The predicted values show only a reasonable agreement due to some restriction of the models. The predicted values mainly depend on the cover and diameter of longitudinal reinforcement and effective tension area.

## 5. Conclusions

The experimental study was conducted to investigate the performance of RC beams strengthened with SNSM-GFRP bars. The following conclusions were made from the experimental program:

- Flexural strengthening of RC beams with the SNSM technique using GFRP bars is effective, as SNSM bars significantly improved the flexural performance via the reduction of the deflection, the delay in the formation of first crack, the decrease in crack width and the increase in the number of cracks and ultimate loads of the specimens compared with the control specimen.
- Strengthening using SNSM-GFRP bars enhanced the first crack and ultimate loads up to 4.38- and 1.55-times compared with the control specimen.
- The use of GFRP as an SNSM reinforcement has exhibited a tri-linear response in load-deflection behavior and reduced the deflection at any load level of the specimens, which would address the serviceability concerns.
- Flexural failure mode was observed in all SNSM-GFRP-strengthened specimens, which is similar to the control specimen. Therefore, the SNSM-GFRP strengthening technique is less prone to debonding.
- Energy absorption capacity, ductility and stiffness under the service load were all significantly enhanced by the SNSM technique via the use of GFRP bars.
- Increasing the bonded length and amount of the strengthening reinforcement improved the flexural performance of the specimens compared with the control specimen. However, the bond length of the strengthened reinforcement has greater influence on the performance compared with the amount of strengthening reinforcement.
- The predicted and experimental results for deflection and flexural crack spacing and the width of the beam specimens are in good agreement.

## Figures and Tables

**Table 1:** Test details for side near surface mounted technique.

Beam ID	Description	Strengthening configuration				
		Materials	Bar diameter (mm)	Bonded length (mm)	Groove size (mm)	Adhesive
CB	Control beam (unstrengthened)					
S1.6D8	RC beams strengthened with the SNSM technique using different amounts of GFRP reinforcements	Glass fiber-reinforced polymer (GFRP) bars	8	1600	25 × 25	Epoxy
S1.7D8				1700		
S1.8D8				1800		
S1.9D8				1900		
S1.6D10			10	1600		
S1.7D10				1700		
S1.8D10				1800		
S1.9D10				1900		

**Table 2:** Properties of GFRP bars.

Diameter (mm)	Density (g/cm^3^)	Ultimate tensile strength (MPa)	Ultimate shear strength (MPa)	E-Modulus (GPa)
8	2.2	1080	150	40
10	2.2	980	150	40

**Table 3:** Summary of the ductility index.

Specimen	$\mu_{\Delta u}$	Compared with CB (%)	$\mu_{\Delta f}$	Compared with CB (%)
CB	1.61	-	3.76	-
S1.6D8	1.41	−12	3.23	−14
S1.7D8	1.56	−3	3.54	−6
S1.8D8	1.85	15	3.75	−0.3
S1.9D8	1.69	5	4.17	11
S1.6D10	1.55	−4	3.74	−0.5
S1.7D10	1.81	12	4.70	25
S1.8D10	1.73	7	4.58	22
S1.9D10	2.23	39	4.75	26
